# Sustaining a Multidisciplinary, Single-Institution, Postoperative Mobilization Clinical Practice Improvement Program Following Hepatopancreatobiliary Surgery During the COVID-19 Pandemic: Prospective Cohort Study

**DOI:** 10.2196/30473

**Published:** 2021-10-06

**Authors:** Kai Siang Chan, Bei Wang, Yen Pin Tan, Jaclyn Jie Ling Chow, Ee Ling Ong, Sameer P Junnarkar, Jee Keem Low, Cheong Wei Terence Huey, Vishal G Shelat

**Affiliations:** 1 Department of General Surgery Tan Tock Seng Hospital Singapore Singapore; 2 Office of Clinical Governance Tan Tock Seng Hospital Singapore Singapore

**Keywords:** enhanced recovery after surgery, early mobilization, liver resection, pancreas surgery, quality improvement project, pancreaticoduodenectomy

## Abstract

**Background:**

The Enhanced Recovery After Surgery (ERAS) protocol has been recently extended to hepatopancreatobiliary (HPB) surgery, with excellent outcomes reported. Early mobilization is an essential facet of the ERAS protocol, but compliance has been reported to be poor. We recently reported our success in a 6-month clinical practice improvement program (CPIP) for early postoperative mobilization. During the COVID-19 pandemic, we experienced reduced staffing and resource availability, which can make CPIP sustainability difficult.

**Objective:**

We report outcomes at 1 year following the implementation of our CPIP to improve postoperative mobilization in patients undergoing major HPB surgery during the COVID-19 pandemic.

**Methods:**

We divided our study into 4 phases—phase 1: before CPIP implementation (January to April 2019); phase 2: CPIP implementation (May to September 2019); phase 3: post–CPIP implementation but prior to the COVID-19 pandemic (October 2019 to March 2020); and phase 4: post–CPIP implementation and during the pandemic (April 2020 to September 2020). Major HPB surgery was defined as any surgery on the liver, pancreas, and biliary system with a duration of >2 hours and with an anticipated blood loss of ≥500 ml. Study variables included length of hospital stay, distance ambulated on postoperative day (POD) 2, morbidity, balance measures (incidence of fall and accidental dislodgement of drains), and reasons for failure to achieve targets. Successful mobilization was defined as the ability to sit out of bed for >6 hours on POD 1 and ambulate ≥30 m on POD 2. The target mobilization rate was ≥75%.

**Results:**

A total of 114 patients underwent major HPB surgery from phases 2 to 4 of our study, with 33 (29.0%), 45 (39.5%), and 36 (31.6%) patients in phases 2, 3, and 4, respectively. No baseline patient demographic data were collected for phase 1 (pre–CPIP implementation). The majority of the patients were male (n=79, 69.3%) and underwent hepatic surgery (n=92, 80.7%). A total of 76 (66.7%) patients underwent ON-Q PainBuster insertion intraoperatively. The median mobilization rate was 22% for phase 1, 78% for phases 2 and 3 combined, and 79% for phase 4. The mean pain score was 2.7 (SD 1.0) on POD 1 and 1.8 (SD 1.5) on POD 2. The median length of hospitalization was 6 days (IQR 5-11.8). There were no falls or accidental dislodgement of drains. Six patients (5.3%) had pneumonia, and 21 (18.4%) patients failed to ambulate ≥30 m on POD 2 from phases 2 to 4. The most common reason for failure to achieve the ambulation target was pain (6/21, 28.6%) and lethargy or giddiness (5/21, 23.8%).

**Conclusions:**

This follow-up study demonstrates the sustainability of our CPIP in improving early postoperative mobilization rates following major HPB surgery 1 year after implementation, even during the COVID-19 pandemic. Further large-scale, multi-institutional prospective studies should be conducted to assess compliance and determine its sustainability.

## Introduction

Enhanced Recovery After Surgery (ERAS) is a multimodal, multidisciplinary perioperative approach to improve surgical outcomes [1]. The implementation of ERAS has improved perioperative outcomes in patients undergoing elective major hepatopancreatobiliary (HPB) surgery [2]. Postoperative early mobilization is an integral component of the ERAS protocols as it reduces pleuropulmonary complications and deep vein thrombosis [3]. Early postoperative mobilization also reduces postoperative ileus and length of hospital stay [4,5]. However, there are no standardized criteria to define mobilization, and compliance remains poor. Vague terminologies, including sitting out of bed, standing at the bedside, walking duration, and walking distances, are used to define mobilization. Recently, Grass et al [6] performed a retrospective study involving 1170 patients who had colorectal surgery in Switzerland to assess early postoperative mobilization (defined as sitting out of bed ≥6 hours on postoperative day [POD] 1). They showed that 58% of patients were noncompliant, with resulting increased postoperative morbidity (overall complications 55% vs 29%, *P*<.001) and length of stay (mean 12, SD 14 days vs mean 6, SD 7 days; *P*<.001) compared to the early mobilization group [6].

A systematic review by Coolsen et al [2] in 2013 described poor compliance (mobilization rate 20%-28%) to early postoperative mobilization on POD 1 following liver surgery [7,8]. Similarly, our institution showed a poor postoperative mobilization rate of 22% in patients undergoing elective major HPB surgery, with improvement to >75% following the implementation of a multidisciplinary surgeon-led clinical practice improvement project (CPIP) [9]. The quality improvement process does not end with the implementation of a solution. Specific steps must be taken, and mechanisms established to hold the gains, for breakthroughs in results come from sustaining changes. The Royal College of Physicians of London, United Kingdom, has incorporated sustainability within the Institute of Medicine’s six quality domains [10]. The median follow-up time for a health care CPIP is reported to be less than 1 year [11]. Only a sustained initiative can be spread for adoption by others at multiple locations so that communities can reap gains.

Ensuring sustainability is difficult due to the COVID-19 pandemic. The COVID-19 pandemic has had a profound impact on the community, health care workers, and health care systems, with more than 3.1 million deaths as of May 2021 [12]. In light of this pandemic, our institution reallocated resources preferentially for COVID-19–related care to cope with clinical demands. Our HPB unit began triaging and scaling down elective surgery to facilitate staff redeployment and reduce patient exposure to the novel coronavirus. Oncology-related services, however, were minimally disrupted given the time-sensitive nature of these diseases and the need for prompt management [13]. Saab et al [14] surveyed 82 centers in 28 countries and described reduced pain management and supportive care services by 26% and limitations in social services support by 74%. To add on, mobilization mandates staff to be near patients, which violates safe distancing measures. A clinical practice guideline by Thomas et al [15] in 2020 for physiotherapy management during the COVID-19 pandemic recommended screening referrals for mobilization and exercise to minimize staff in contact and high-filtration masks during physiotherapy sessions. Locally, personal protective equipment was mandatory for physiotherapists, and ambulation was limited to the patients’ ward cubicle to minimize external contact. There are also increased stressors associated with fear and anxiety of becoming infected [16]. Hence, this study aimed to assess the sustainability of our multidisciplinary single-institution CPIP at 1-year postimplementation to improve the postoperative mobilization rate of patients undergoing elective major HPB surgery during the COVID-19 pandemic.

## Methods

### Overview

Our institution is a university-affiliated tertiary hospital with 1700 inpatient beds. ERAS started in March 2016 in the colorectal surgery division. In line with the concept of ERAS, the HPB unit began inpatient prehabilitation for patients undergoing elective liver surgery in 2016, resulting in a reduction in overall morbidity and improved social well-being [17]. The entire ERAS protocol subsequently expanded to the HPB unit for patients undergoing elective major HPB surgery in 2018. The HPB surgery dashboard for 2018 following the implementation of ERAS revealed a low observed/expected ratio for compliance, with a postoperative mobilization rate of 22%. Hence, relevant stakeholders agreed to implement a CPIP to improve postoperative mobilization, which began in May 2019 [9].

### Study Protocol

The specific details of our CPIP were described in 2020 by Tang et al [9]. Successful mobilization was defined as sitting out of bed for >6 hours on POD 1 and ambulation of ≥30 m on POD 2, with a target mobilization rate of ≥75%. Preoperatively, case managers counsel patients and caregivers on postoperative goals and emphasize the benefits of early mobilization. Postoperatively, the surgical teams emphasize the benefits of mobilization during POD 1 evening rounds. The plan-do-study-act (PDSA) cycles were utilized to identify critical barriers to early mobilization, and changes were implemented to identify outcomes. Major HPB surgery was defined as surgery involving the HPB system and lasting more than 2 hours. Patients were excluded from the study if they had undergone cholecystectomy, common bile duct exploration, laparotomy for general surgical conditions, or major HPB surgery with intraoperative blood loss of ≥2 L or a surgery duration of >9 hours.

We summarized the entire mobilization improvement process into 4 phases:

- Phase 1 (January to April 2019): prior to CPIP implementation;
- Phase 2 (May to September 2019): CPIP implementation, where there is direct oversight to improve postoperative mobilization using the PDSA cycles;
- Phase 3 (October 2019 to March 2020): post-CPIP, before the COVID-19 pandemic, where there was indirect oversight of postoperative mobilization. This was also required routinely as part of our institution’s protocol following CPIP implementation;
- Phase 4 (April 2020 to September 2020): post-CPIP, during the COVID-19 pandemic, where there was no oversight on interventions to improve postoperative mobilization.

**Figure 1:**
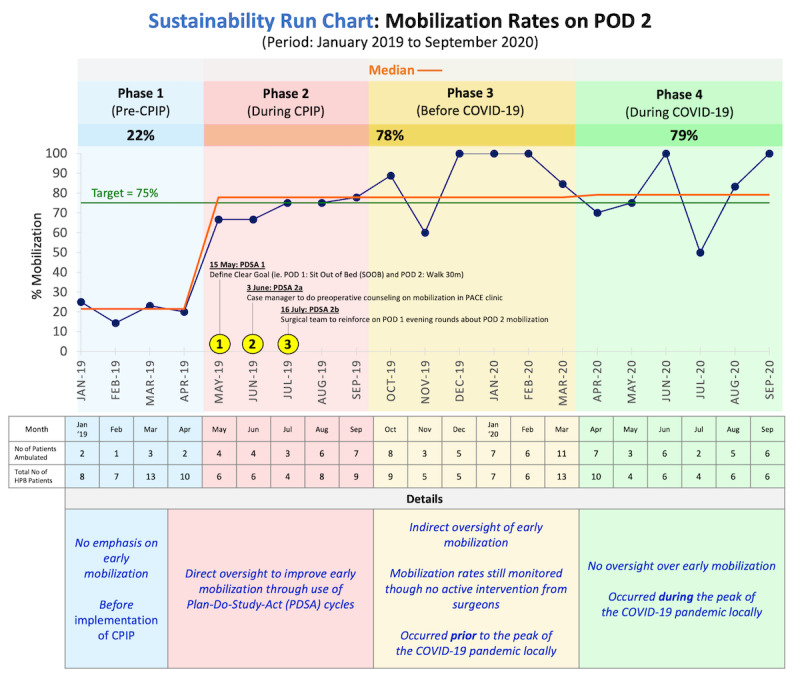
Schematic diagram of the 4 phases of the improvement of early postoperative mobilization and their respective time frames, monthly mobilization rates on postoperative day (POD) 2, and median mobilization rates during different phases. CPIP: clinical practice improvement program, PACE: Pre-Admission Counselling and Evaluation, HPB: hepatopancreatobiliary.

Figure 1 is a schematic representation of the 4 phases of the mobilization improvement process. As the purpose of this study is to assess the sustainability following our CPIP during the COVID-19 pandemic, we will be primarily describing phase 4 of our study.

### Impact of COVID-19 Locally

The first case of COVID-19 in Singapore was detected in January 2020, and the national “circuit breaker measures” were announced on April 7, 2020 [18]. This gave us the chance to stratify the analysis into phase 3 (before COVID-19, from October 2019 to March 2020) and phase 4 (during COVID-19, from April 2020 to September 2020). During phase 4, there were three modifications to postoperative mobilization due to COVID-19: (1) physiotherapists had to wear full personal protective equipment during physiotherapy sessions, (2) mobilization was limited to within the patients’ cubicles to limit the risk of infection, and (3) segregation of physiotherapy teams to reduce cross-contact among the health care personnel.

### Perioperative Management and Prehabilitation Program

In 2016, the HPB unit introduced an inpatient prehabilitation program, 2 to 4 weeks in duration [17]. The program involves a multidisciplinary team comprising physiotherapists, dieticians for nutrition optimization, and case managers for patient education on the surgery and postoperative expectations. All elective major HPB surgical patients were considered for the program unless excluded due to logistic reasons or resource constraints (eg, the surgery date was too close or there was a lack of program slots). Pain management is an integral part of the prehabilitation program and an essential component relevant to mobilization. We adopted a multimodal approach to manage postoperative pain; the ON-Q PainBuster (B Braun Melsungen AG), an elastomeric pump device providing a continuous infusion of 400 ml of ropivacaine, was intraoperatively inserted at the discretion of the surgeon in the preperitoneal space. The ON-Q PainBuster was not routinely inserted for laparoscopic procedures. The majority of patients were also on patient-controlled analgesia (PCA) (fentanyl) and paracetamol postoperatively. Epidural analgesia is infrequently used at our institution in view of the following reasons: (1) placement and removal of epidural analgesia is more technically challenging and slower compared to the ON-Q PainBuster; (2) recommendations from the American Society of Regional Anesthesia that an international normalized ratio (INR) of 1.4 is the upper limit for safe removal of an epidural catheter (in our group of patients undergoing major hepatic resections, an abnormal INR is to be expected and would require fresh frozen plasma coverage [19]); and (3) local protocol mandating the need for monitoring in high-dependency units while on epidural analgesia (this precludes patients who are clinically improving and stable after transfer to the general ward and occupy limited high-dependency beds for patients who may require high-dependency monitoring).

### Data Collection and Study Variables

Data were extracted for all patients included in this study from phases 2 to 4 (May 2019 to September 2020) from a prospectively maintained standing database for patients undergoing HPB surgery approved by the local institutional review board. Data were not extracted for patients for phase 1 (before CPIP implementation). Study variables included insertion of the ON-Q PainBuster intraoperatively, pain score, length of hospital stay, distance ambulated on POD 2, morbidity, balance measures, and reasons for failure to achieve targets. Length of hospitalization stay was defined as the duration of hospital stay calculated from admission to the point of discharge. Successful mobilization was defined as sitting out of bed for >6 hours on POD 1 and ambulation of ≥30 m on POD 2 [9]. Morbidity was defined as the incidence of pneumonia and deep vein thrombosis. Balance measures, defined as potential complications secondary to mobilization—incidence of falls and accidental drain dislodgement—were evaluated.

### Statistical Analysis

All data were tabulated into an Excel sheet (Microsoft Corp) and transposed into SPSS, version 25.0 (IBM Corp), for statistical analysis. Categorical values were described as percentages and analyzed by the chi-square test and Fisher exact test for variables with expected cell count <5. Continuous variables were described as median (IQR) or mean (SD) and were analyzed by the Kruskal-Wallis test or analysis of variance (ANOVA), respectively. Statistical significance was defined as a *P* value of <.05.

## Results

### Baseline Demographics and Clinical Profile

A total of 114 patients underwent major HPB surgery from phases 2 to 4 of our study, with 33 (29.0%), 45 (39.5%), and 36 (31.6%) patients in phases 2, 3, and 4, respectively. Baseline patient demographic data were not collected for phase 1 (pre–CPIP implementation); information on the median mobilization rate of 22% during phase 1 was obtained from the HPB surgery dashboard in 2018 following the implementation of the ERAS protocol in our HPB unit. The majority of the patients were male (n=79, 69.3%) and underwent hepatic surgery (n=92, 80.7%). There were 76 (66.7%) patients who had an ON-Q PainBuster insertion intraoperatively. Table 1 summarizes the demographics of the study population from phases 2 to 4.

**Table 1:** Patient demographics and clinical profile of the study population.

Characteristic		Overall cohort^a^ (n=114)		Phase 2^b^ (n=33)		Phase 3^c^ (n=45)		Phase 4^d^ (n=36)	*P* value
Age (years), median (IQR)		66.5 (60.8-71.3)		67 (56-71)		66 (61-71)		67 (61.3-72)	.74
Gender (male), n (%)		79 (69.3)		20 (60.6)		31 (68.9)		28 (77.8)	.30
**ASA^e^ score, n (%)**									.19
	I	1 (0.9)	1 (3.0)		0 (0)		0 (0)		
	II	38 (33.3)	11 (33.3)		19 (42.2)		8 (22.2)		
	III	75 (65.8)	21 (63.6)		26 (57.8)		28 (77.8)		
Prehabilitation, n (%)		63 (55.3)		21 (63.6)		19 (42.2)		23 (63.9)	.08
**Surgical approach, n (%)**									.01
	Laparoscopic	40 (35.1)	13 (39.4)		12 (26.7)		15 (41.7)		
	Laparoscopic converted open	5 (4.4)	0 (0)		0 (0)		5 (13.9)		
	Open	69 (60.5)	20 (60.6)		33 (73.3)		16 (44.4)		
**Type of surgery, n (%)**									.42
	Hepatic	92 (80.7)	25 (75.8)		39 (86.7)		28 (77.8)		
	Pancreatic	22 (19.3)	9 (24.2)		6 (13.3)		8 (22.2)		
Placement of ON-Q PainBuster (yes), n (%)		76 (66.7)		20 (60.6)		33 (73.3)		23 (63.9)	.46
**Abdominal drains, n (%)**									.49
	0	31 (27.2)	12 (36.4)		13 (28.9)		6 (16.7)		
	1	69 (60.5)	17 (51.5)		27 (60.0)		25 (69.4)		
	2	13 (11.4)	4 (12.1)		4 (8.9)		5 (13.9)		
	3	1 (0.9)	0 (0)		1 (2.2)		0 (0)		

^a^Overall cohort refers to the study population from phases 2 to 4. Study demographics are not shown for phase 1 patients.

^b^Phase 2 refers to the period during CPIP implementation (May 2019 to September 2019).

^c^Phase 3 refers to the sustainability phase post-CPIP but before the COVID-19 pandemic (October 2019 to March 2020).

^d^Phase 4 refers to the sustainability phase post-CPIP during the COVID-19 pandemic (April 2020 to September 2020).

^e^ASA: American Society of Anesthesiologists.

### Postoperative Outcomes

Table 2 summarizes the outcomes of the study population in each phase of the study.

The median mobilization rate was 22% for phase 1, 78% for phases 2 and 3 combined, and 79% for phase 4 (Figure 1). We combined the median mobilization rate for phases 2 and 3 as the mobilization rate at the start of phase 2 will be lower since it takes time for the effects of the CPIP to be seen; in April 2019 (prior to the start of phase 2), mobilization was 2 out of 10 (20%). The mean pain score was 2.7 (SD 1.0) on POD 1 and 1.8 (SD 1.5) on POD 2. A pairwise comparison of pain score on POD 2 showed a significant difference in pain score between phases 2 and 4 (phase 2: pain score 2.3, SD 1.8 vs phase 4: pain score 1.3, SD 1.3; *P*=.01). The median length of hospital stay was 6 days (IQR 5-11.8). There were no falls or accidental dislodgement of drains. A total of 6 patients (5.3%) had pneumonia.

**Table 2:** Study population outcomes.

Characteristic		Overall cohort^a^ (n=114)	Phase 2^b^ (n=33)	Phase 3^c^ (n=45)	Phase 4^d^ (n=36)	*P* value	
Operating time (min), median (IQR)		338 (240-489)	248 (178-379)	285 (228-353)	350 (259-456)	.01	
Blood transfusion (yes), n (%)		20 (17.5)	5 (15.2)	6 (13.3)	9 (25.0)	.36	
Length of hospital stay (days), median (IQR)		6 (5-11.8)	6 (4-8)	6 (5-8)	6.5 (4-17)	.79	
**Pain score, mean (SD)**							
	POD^e^ 1	2.7 (1.0)	2.7 (0.7)	2.7 (1.0)	2.7 (1.5)	.98	
	POD 2	1.8 (1.5)	2.3 (1.8)	1.8 (1.2)	1.3 (1.3)	.01	
**Ambulated ≥30 m on POD 2 (yes), n (%)**		93 (81.6)	24 (72.7)	40 (88.9)	29 (80.6)	.19	
	Actual distance walked (m), median (IQR)	50 (30-100)	40 (21-100)	70 (45-100)	50 (30-100)	.16	
**Reasons for failing to achieve target ambulation (n=21), n (%)**							.51
	Pain	6 (28.6)	2 (22.2)	2 (40.0)	2 (28.6)		
	Lethargy/giddiness	5 (23.8)	3 (33.3)	1 (20.0)	1 (14.3)		
	Nausea	2 (9.5)	1 (11.1)	1 (20.0)	0 (0)		
	Hypotension/tachycardia	2 (9.5)	1 (11.1)	0 (0)	1 (14.3)		
	Medical instructions (postchest tube removal)	2 (9.5)	2 (22.2)	0 (0)	0 (0)		
	Local protocols (ongoing blood transfusion)	2 (9.5)	0 (0)	1 (20.0)	1 (14.3)		
	Admitted to ICU^f^	2 (9.5)	0 (0)	0 (0)	2 (28.6)		
**Morbidity, n (%)**							
	Falls	0 (0)	0 (0)	0 (0)	0 (0)	N/A^g^	
	Pneumonia	6 (5.3)	2 (6.1)	1 (2.2)	3 (8.3)	.46	
	Deep vein thrombosis	0 (0)	0 (0)	0 (0)	0 (0)	N/A	

^a^Overall cohort refers to the study population from phases 2 to 4. Study demographics are not shown for phase 1 patients.

^b^Phase 2 refers to the period during CPIP implementation (May 2019 to September 2019).

^c^Phase 3 refers to the sustainability phase post-CPIP but before the COVID-19 pandemic (October 2019 to March 2020).

^d^Phase 4 refers to the sustainability phase post-CPIP during the COVID-19 pandemic (April 2020 to September 2020).

^e^POD: postoperative day.

^f^ICU: intensive care unit.

^g^N/A: not applicable.

### Reasons for the Failure of Early Postoperative Mobilization

Table 2 summarizes the reasons for the failure of early postoperative mobilization. A total of 21 patients (18.4%) failed to ambulate ≥30 m on POD 2. Among these patients, 15 (71.4%) underwent open surgery, and 17 (81.0%) had the ON-Q PainBuster inserted intraoperatively. A total of 13 patients (61.9%) had either inpatient or outpatient prehabilitation before the surgery. The most common reason for failure to achieve the ambulation target was pain (6/21, 28.6%), followed by lethargy or giddiness (5/25, 23.8%). In addition, 2 patients (9.5%) were required to have complete rest in bed due to chest tube removal, and 2 patients (9.5%) had ongoing blood transfusions upon review by the physiotherapist and hence did not ambulate. Another 2 patients (9.5%) were admitted in the intensive care unit and were not stable enough for physiotherapy.

## Discussion

### Principal Findings

Our study demonstrated the long-term sustainability of the CPIP to promote early mobilization following elective major HPB surgery after CPIP implementation. The mobilization rate during the COVID-19 pandemic was 79%.

CPIPs target a specific, measurable goal, identify critical barriers, and develop a model for improvement. We previously described the success of our CPIP in improving postoperative mobilization [9]. A quality dashboard inclusive of a Pareto chart was provided to clinician stakeholders in 2018. Engagement of physiotherapy and nursing colleagues was done to understand the micro and macro workflows relevant to mobilization. Root cause analysis for barriers to mobilization was done by a core team trained in CPIP use. The surgeon-led multidisciplinary quality improvement initiative with multiple PDSA cycles adhering to the CPIP philosophy led to improved process outcomes along with cost savings [9]. However, a CPIP can only be successful if it is sustainable. Sustainability is defined as the capacity of a health service to deliver health care over time with considerations for future generations [10]. It is an essential facet of health care innovation. It has, therefore, been incorporated to be included in the Institute of Medicine’s six domains of quality by the Royal College of Physicians [10]. Alexander et al [11] concluded that the median follow-up time for health care quality improvement projects was less than 1 year, which is insufficient to observe the long-term effects of any implementation on clinical outcomes. We continued the follow-up of our CPIP for 1 year following the implementation and divided our analysis into phase 3 (before COVID-19) and phase 4 (during COVID-19) to observe differences during these two periods. During both periods, we achieved a ≥75% target mobilization rate.

Early mobilization is a facet of the ERAS program; a meta-analysis by Ji et al [20] on the use of ERAS in pancreatic surgery demonstrated lower incidence of delayed gastric emptying (odds ratio (OR) 0.58, 95% CI 0.48-0.72, *P*<.001), lower postoperative complication rates (OR 0.57, 95% CI 0.45-0.72, *P*<.001), and shorter length of hospital stay (weighted mean difference –4.45, 95% CI –5.99 to –2.91, *P*<.001). However, compliance was not reported in the meta-analysis. The failure of ERAS programs may be due to a lack of compliance rather than the concept of ERAS itself [21,22]. Elias et al [23] published the Reporting on ERAS Compliance, Outcomes, and Elements Research (RECOvER) checklist to improve compliance, including the need to describe a strategy for early mobilization. They defined early mobilization as fulfilling all of the following: (1) in the postoperative anesthesia unit, to ambulate from bed to chair, (2) on POD 0, to ambulate 3 times and sit out of bed for all meals (no distance or time duration specified), and (3) on POD 1, to sit out of bed for ≥8 hours. This provides a standardized checklist with a clear definition, but the definition of early mobilization is heterogeneous in other studies. Wind et al [24] defined early mobilization as sitting out of bed for >2 hours on POD 0, >6 hours on POD 1, and >8 hours on POD 2. Gatt et al [25] defined it as sitting out of bed on POD 0 and ambulating the length of the ward on POD 1. We defined early mobilization as sitting out of bed for >6 hours on POD 1 and ambulating ≥30 m on POD 2. A review of existing literature on ERAS programs showed heterogenous definitions of early postoperative mobilization, ranging from “time spent out of bed” and “time ambulated” to “distance or steps walked on POD 2 or beyond.” Hence, the value of “30 m” was chosen based on past experience and the practical needs of patients in our local context: 30 m is the approximate distance to ambulate from the living room to the toilet and back. The ability to do so would suggest that the patient is able to independently carry out activities of daily living, making this a meaningful distance target. Furthermore, the to-and-fro distance from patients’ cubicle to the ward entrance is approximately 30 m, making it logistically easier for physiotherapists and nurses to record the distance ambulated [9,26]. It is important to note that the terminologies “mobilization” and “ambulation” are not synonymous. Patients were required to fulfill both criteria—sitting out of bed for >6 hours on POD 1 and ambulating ≥30 m on POD 2—to be deemed successful in early postoperative mobilization. While we agree that patients are instructed to sit out of bed usually on either POD 0 or POD 1, it is the act of ambulating that is more relevant to patients’ physiologic function and activities of daily living. Therefore, we defined mobilization by the act of ambulating, rather than by only sitting out of bed. Further prospective studies examining postoperative mobilization should use standardized and concise definitions of mobilization to have a clear endpoint and for results to be reproducible for large-volume meta-analyses.

The COVID-19 pandemic has resulted in disruption in the delivery of health care services, especially in the surgical subspecialties. Recommendations were made for postponing elective surgical cases where possible [27]. Locally, there was a shift toward nonoperative management for stable, benign conditions such as uncomplicated acute cholecystitis [28]. This was to redirect resources toward the management of patients with COVID-19. Our study, however, showed that we were able to maintain mobilization targets even during the pandemic. Following the CPIP, we continued implementing preoperative counseling and reinforcing the importance of early mobilization on POD 1 during routine ward rounds. Reasons that were previously identified for failure to ambulate continue to be addressed. Pain was the most common reason for failure to achieve the ambulation target (n=6, 28.6%). In our institution, the ON-Q PainBuster was placed intraoperatively in the preperitoneal space for major open surgeries to deliver bupivacaine or ropivacaine through continuous infusion. This is reported to be effective in reducing postoperative pain and facilitating early ambulation compared to a placebo [29]. PCA was also used as part of our multimodal approach for analgesia. Nevertheless, pain remained the most typical reason for failure to achieve ambulation targets; this is likely because of the need to balance the side effects of excessive analgesic use, such as nonsteroidal inflammatory drugs, with the risk of renal impairment adverse cardiac events and gastrointestinal bleeding [30]. Use of opioids is also associated with delayed recovery of bowel function, as well as postoperative nausea and vomiting, which may limit ambulation. Therefore, titration of analgesia needs to obtain the best control of pain and limit side effects to improve mobilization. Interestingly, there was a significant reduction in pain score on POD 2 from 2.3 (SD 1.8) during phase 2 to 1.3 (SD 1.3) during phase 4 (*P*=.01), with comparable incidence of laparoscopic surgery, which may have contributed to the sustainability of early postoperative mobilization. While improved pain incentivizes patients to mobilize early, Ni et al [31] demonstrated improved pain scores on POD 5 in patients who had early ambulation compared to the control group (mean 3.1, SD 1.1 vs mean 3.8, SD 2.4; *P*<.05). To add on to the discussion, while epidural analgesia is an alternative for pain control, our institution prefers ON-Q PainBuster to epidural infusion as ON-Q PainBuster is relatively easier to insert and remove and does not require an INR ≤1.4 for safe removal unlike epidural catheter. A systematic review by Mungroop et al [32] showed that preperitoneal wound catheters provide statistically, but not clinically, significantly different pain control at rest on POD 1 as epidural analgesia (mean difference 0.44, 95% CI 0.06-0.79; *P*=.02), with a lower incidence of hypotension (relative risk 0.29, 95% CI 0.13-0.68; *P*=.004) and patient satisfaction. While pain is the most common reason for failure of ambulation, we have attempted to mitigate it via adoption of a multipronged pain control approach.

Further, a plausible reason for the sustainability in having high postoperative mobilization rates despite no active oversight could be due to staff empowerment following CPIP implementation. Our CPIP has emphasized the importance of early postoperative mobilization, with the aim of ambulating ≥30 m on POD 2. Chong et al [33], who studied nurses’ practices regarding early mobilization among mechanically ventilated patients, found that the majority of nurses (99.2%) observed in-bed mobilization among patients, but only a minority (14.4%) saw out-of-bed mobilization. They attributed the lack of doctors’ order for physiotherapy or the lack of nursing staff availability as possible reasons for the lack of out-of-bed mobilization [34]. In line with this, we feel that the strong reinforcement of early postoperative mobilization has provided nursing staff with confidence to allow patients to sit out of bed on POD 1 and promote early mobilization where feasible and when permitted by resource availability. Other indirect measures played by nursing colleagues include charting of pain scores and provision of adequate analgesia to manage pain, which is the most common factor for the lack of early postoperative mobilization [35].

Early postoperative mobilization has been shown to improve clinical outcomes, with a reduced length of hospital stay and incidence of pneumonia and deep vein thrombosis [2,3]. Incidence of postoperative pneumonia following liver surgery has been reported to range from 8.2% to 13% [36-39]. Mobilization has been postulated to elicit cardiopulmonary responses, which enhance oxygen transport, increase tidal volume that may reverse atelectasis, and improve gas exchange or reduce the risk of aspiration in view of an upright position [40]. Our study showed a relatively lower 5.3% overall incidence of postoperative pneumonia from phases 2 to 4, which may be multifactorial: laparoscopic surgery, prehabilitation, early mobilization, and multimodal analgesia with adequate pain control [41].

To improve clinical outcomes, it is integral to improve the process outcomes of all integral components of ERAS protocols. Increasing compliance to existing protocols is an important step forward [2]. Our study demonstrated improvement in early postoperative mobilization rates within our institution; however, our sample size is relatively small, and the generalizability of the results is limited due to the heterogeneous patient population. The concept and technology of health information exchange (HIE) may be adopted to improve the situation. HIE as defined as the use of technology to share clinical and administrative data electronically across health care institutions and repositories; it may be considered to facilitate large-scale prospective studies to provide improved quality of health care and cost savings [42]. A novel method of tracking the compliance and development of predictive risk scores for various clinical outcomes was recently developed by Cochran et al [43]. Research Electronic Data Capture (REDCap), which is an electronic data management system primarily used for data collection, was used to track compliance to ERAS protocols in our institute. Use of both health informatics and REDCap simplifies the process of tracking clinical outcomes and disseminating clinical performance indicators. This permits a quick update of the ERAS dashboard, planning of targeted interventions to improve outcomes, and easy sharing of data across institutions through the HIE technology. Furthermore, embracing these technologies reduces missing data and recording bias to some extent. Institutions with ongoing ERAS protocols should also re-examine their respective surgery dashboards to ensure continued quality improvements. Interinstitutional collaboration should also be encouraged to facilitate high-powered evidence.

### Strengths and Limitations

The strength of our study is that it is, to our knowledge, the only study to report the long-term sustainability of mobilization during the COVID-19 pandemic. We also included reasons for failure to achieve ambulation targets to improve future CPIPs. However, this study has a few limitations. Heterogeneity of the study population, which includes patients who underwent liver surgery and pancreatic surgery, limits the generalizability of the results. We also did not assess the benefits of early postoperative mobilization on clinical outcomes, such as length of hospitalization and postoperative morbidity [2]. The primary aim of this study was descriptive, to describe our experience in sustaining the CPIP at 1 year following implementation during the COVID-19 pandemic. This study also included patients who underwent prehabilitation, which may indirectly have led to the achievement of the mobilization target.

### Conclusion

This follow-up study demonstrated the sustainability of our CPIP in improving early postoperative mobilization rates in patients who underwent elective major HPB surgery 1 year following implementation, even during the COVID-19 pandemic. Further large-scale, multi-institutional prospective studies are needed to define mobilization and assess compliance to early mobilization initiatives. Sustaining a clinical improvement initiative is an essential determinant of value-driven patient-centric health care.

## Abbreviations

ANOVA: analysis of variance
CPIP: clinical practice improvement program
ERAS: Enhanced Recovery After Surgery
HIE: health information exchange
HPB: hepatopancreatobiliary
INR: international normalized ratio
OR: odds ratio
PCA: patient-controlled analgesia
PDSA: plan-do-study-act
POD: postoperative day
RECOvER: Reporting on ERAS Compliance, Outcomes, and Elements Research
REDCap: Research Electronic Data Capture
